# Survival prediction among patients with non-cancer-related end-stage liver disease

**DOI:** 10.1371/journal.pone.0202692

**Published:** 2018-09-21

**Authors:** Yi-Wen Tsai, I-Shiang Tzeng, Yi-Chuan Chen, Tsung-Han Hsieh, Shy-Shin Chang

**Affiliations:** 1 Department of Family Medicine, Chang Gung Memorial Hospital, Keelung, and Chang Gung University College of Medicine, Taoyuan, Taiwan; 2 Department of Research, Taipei Tzu Chi Hospital, Buddhist Tzu Chi Medical Foundation, New Taipei City, Taiwan; 3 Department of Family Medicine, Chang Gung Memorial Hospital, Linkou, and Chang Gung University College of Medicine, Taoyuan, Taiwan; 4 Tissue Bank, Chang Gung Memorial Hospital, Taoyuan, Taiwan; 5 Department of Family Medicine, Taipei Medical University Hospital, Taipei City, Taiwan; 6 Graduate Institute of Clinical Medical Sciences, College of Medicine, Chang Gung University, Taoyuan, Taiwan; University of North Carolina at Chapel Hill School of Medicine, UNITED STATES

## Abstract

**Background:**

Predicting the survival of non-cancer related end-stage-liver-disease patients in general practice has been difficult for physicians because of the extremely variable trajectories due to multiple complex clinical factors, hence it remains a challenging issue to date. This study aimed to develop and validate a specific prognostic scoring system to early recognize the prognosis and improve the quality of end-of life care for non-cancer end-stage-liver-disease population.

**Materials and methods:**

A multicentre, retrospective cohort study was conducted during January 2010 ~ December 2012 and continued follow-up until December 2014. A cox proportional hazard regression analysis was used to derive and validate an optimized model. The main outcome measures were the 28-day, 3-month, 6-month, and 12-month mortality prediction. The performance of the novel model was evaluated, including discrimination and calibration.

**Results:**

A total of 4,080 consecutive subjects were enrolled. The AUROCs for the 3-month survival discrimination in the MELD, MELD-Na and novel model were 0.787, 0.705 and 0.804 (P<0.001); the 6-month survival discrimination were 0.781, 0.702 and 0.797 (P<0.001); the overall survival discrimination were 0.771, 0.694 and 0.785 (P = 0.002) respectively, whereas the novel model showed a significantly higher discrimination power than did the MELD and MELD-Na for the 3-month, 6-month and overall survival prediction. In addition, calibration of external validation cohort showed no statistical difference in all 5 groups compared with the observed groups.

**Conclusion:**

This is a clinically relevant, validated scoring system that can be used sequentially to stratify the prognosis in non-cancer cirrhotic populations, which may help the patients along with medical team in decision making to improve the quality of end-of-life care.

## Introduction

The high mortality from end-stage liver disease (ESLD) is a global public health problem. Early identification of people at risk of downslope prognosis is fundamental for decision making in the clinical settings. Several randomized controlled trials have shown that application of palliative care at least 6 months before death can improve the symptoms, reduce unplanned hospital admissions, minimize aggressive cancer treatments, and hence improve the quality of end-of-life care in this population[1–4]. According to a survey conducted in England, around three-fourths of the population died due to chronic conditions, such as end-stage brain, heart, lung, kidney, and liver diseases, and the ratio of cancer to non-cancer deaths was about 1:2, among which up to 69–82% cases need end-of-life care planning[5, 6].

Although the demand of end-of-life care planning in non-cancer patients is high, non-cancer patients seldom receive end-of-life care planning in time compared with cancer patients[7]. For example, in Taiwan, less than 0.4% of non-cancer patients received end-of-life care planning before they died, and the percentage was by far lower than that (almost 40%[8]) of terminal cancer patients. One of the reasons that caused the low percentage of end-of-life care planning initiation in terminally ill non-cancer patients was the extremely variable trajectory among patients because of complex clinical factors[9, 10]. Therefore, early recognition of prognosis in these patients is important.

Many prognostic models have been proposed in the past decades in patients with chronic liver disease (CLD) for different purposes[11–13]. Among these models, the Child—Pugh score (C-P score), the Model for End-Stage Liver Disease (MELD), and the Model for End-Stage Liver Disease-Sodium (MELD-Na) score are the most widely used tools. Nevertheless, limitations were observed when applying these models to the generalized CLD populations. For example, severity of two component of the C-P scores, ascites and encephalopathy, were subjective variables and each component was given with the same weight in the scoring system[12, 14, 15]. The MELD and MELD-Na score, although commonly applied as an objective scale for disease severity, were used mostly for short-term mortality prediction, aiming at predicting survival after transjugular intrahepatic portosystemic shunt placement[16–18].

To date, only a few prognostic scoring systems are available for earlier survival prediction in non-cancer end-stage liver disease patients. Therefore, this study aimed to develop a prognostic model to aid the clinician in decision making in end-of-life care. Second, an external validation set was used to validate this novel model.

## Materials and methods

### Study subjects and data collection

A multicentre, retrospective cohort study was conducted using data obtained upon admission and from the outpatient department of the three branches of Chang Gung Memorial Hospital at Linkou, Taipei, and Keelung between January 2010 and December 2012; follow-up was continued until December 2014. The validation data set was obtained between 2013 and 2014. Data sets were collected from electronic medical records (EMRs) of patients aged ≥18 years diagnosed with cirrhosis (ICD-9-CM code 571.xx) with or without related complications of hepatic coma (ICD-9-CM code 070.xx; 572.xx), spontaneous bacterial peritonitis (ICD-9-CM code 567.xx), oesophageal varices (ICD-9-CM code 456.xx), and sepsis (ICD-9-CM code 995.xx; 785.xx; 038.xx). Individuals meeting the above criteria and whose laboratory data of EMRs were available within 24 hours of admission were included in this study. The exclusion criteria were patients who were diagnosed with malignancy, pregnancy, or liver transplantation.

This study was conducted in accordance with the ethical standards of the Helsinki Declaration, and both the derivation and the validation data sets were based on studies approved by the Ethical Review Boards of Chang Gung Memorial Hospital. Approval number: 103-7785B.

### Measurements and definitions

Data for calculating the MELD score and MELD-Na score and other biochemistry data associated with CLD, including complete blood counts (CBC), prothrombin time (PT), alanine transaminase (ALT), aspartate transaminase (AST), serum albumin, blood urine nitrogen (BUN), creatinine (Cr.), total bilirubin, serum sodium, serum potassium, and international normalized ratio (INR), were collected. Patients’ demographic and clinical characteristics, including age, sex, and aetiology of CLD, such as viral hepatitis carrier or alcoholic hepatitis, were also recorded.

The MELD score was calculated according to the following formula: MELD score = 3.78 × log_*e*_(Bilirubin [mg/dL]) + 11.2 × log_*e*_(INR) + 9.57 × log_*e*_(Creatinine[mg/dL]) + 6.43 [19]. The MELD-Na score was calculated according to the following formula: MELD—Na = MELD + 1.59 × (135 –serum sodium), where the serum sodium concentration is bound between 120 and 135 mmol/L[20].

Mortality was defined as coding of EMRs with death or critical against medical advice discharge.

### Study outcomes and statistical analysis

The main outcome measure was to develop a novel model for survival prediction and to compare its performance to predict the 28-day, 3-month, 6-month, and 12-month mortality using the MELD and MELD-Na scores in non-cancer CLD populations. Moreover, the validation of the novel model was further examined.

The differences of prognostic factors of non-cancer end-stage liver disease with and without mortality groups were compared during the follow-up period. Next, stepwise Cox proportional hazards regression was used to construct the novel model. The concordance (c-statistic) equivalent to the area under the receiver operating characteristic curve (AUROC) was measured to examine the performance of the novel model to discriminate the 28-day, 3-month, 6-month, and 12-month mortality. The ability of mortality prediction of the novel model was further compared with the MELD and MELD-Na scores. Moreover, a validation cohort was constructed to verify the external utility of the new model to a heterogeneous group of patients. The continuous variables were analysed using the median test. In addition, the continuous variables of clinical data were converted into categorical variables by cut-off points which refer to clinical practice, protocols in other studies, or associated laboratory references. To analyse the categorical variables, a Chi-square test was used. Last, the calibration test of the external validation data set was also performed.

All the statistical analyses were performed using R (R, version 3.3.2, Vienna, Austria 2008) software. For all analyses, a P value of <0.05 was regarded to suggest statistical significance.

## Results

### Comparison of demographic and laboratory characteristics between cirrhotic patients with and without mortality during the follow-up period

A total of 4,080 cirrhotic patients were enrolled in this study. During the follow-up period between 2010 and 2014, the overall mortality rate was 29.1% (n = 1,188). Table 1 shows the difference of demographic and laboratory characteristics in cirrhotic patients with and without mortality during the observation period. The mean ages were 53 (43, 64) years and 59 (48, 72) years, and the numbers (percentage) of male patients were 1,997 (69.1%) and 822 (69.2%) in the non-mortality and mortality groups. No statistical difference was observed in the prevalence of different aetiologies of cirrhosis between the two groups (21.4%, 18.3%, and 30.8% for chronic hepatitis B infection, hepatitis C infection, and alcoholic hepatitis, respectively, in the non-mortality group as compared with 23.3%, 15.8%, and 33.6% in the mortality group, respectively). Nevertheless, the prevalence of cirrhotic complications, such as spontaneous bacterial peritonitis (SBP) [n = 203 (7.02%) vs. n = 213 (17.9%), p<0.001], hepatoencephalopathy [n = 369 (12.8%) vs. n = 472 (39.7%), p<0.001], bleeding oesophageal varices (EV) [n = 654 (22.6%) vs. n = 350 (29.5%), p<0.001], and sepsis [n = 390 (13.5%) vs. n = 532 (44.8%), p < 0.001], was found to have statistically significant difference between the groups with and without mortality. In addition, the biochemistry profiles, such as platelet counts, PT, ALT, AST, total bilirubin, serum sodium level, creatinine (Cr.), international normalized ratio (INR), albumin level, etc., all reached significant difference in the two groups. Compared with the non-mortality group, those in the mortality group were older, with lower platelet counts; more prolong PT; higher level of serum ALT, total bilirubin, Na, Cr., and INR; and lower level of serum albumin (all p < 0.001).

**Table 1:** Comparison of demographic and laboratory characteristics of all non-cancer cirrhotic patients with and without mortality during the follow-up period between 2010–2014. (N = 4080).

Characteristics	Non-mortality	Mortality	p Value
	(n = 2892, 70.9%)	(n = 1188, 29.1%)	
**Age (years)**	53 (43, 64)	59 (48, 72)	<0.001*
**Gender, n (%)**			0.960
Female	895 (30.9)	366 (30.8)	
Male	1997 (69.1)	822 (69.2)	
**Etiology, n (%)**			
HBV	619 (21.4)	277 (23.3)	0.204
HCV	528 (18.3)	188 (15.8)	0.070
Alcohol	892 (30.8)	399 (33.6)	0.094
**Complicatios, n (%)**			
SBP	203 (7.02)	213 (17.9)	<0.001 *
Hepatoencephalopathy	369 (12.8)	472 (39.7)	<0.001 *
EV bleeding	654 (22.6)	350 (29.5)	<0.001 *
Sepsis	390 (13.5)	532 (44.8)	<0.001 *
**WBC (10**^**3**^**/uL)**	7.06 (4.3, 8.5)	9.55 (4.9, 11.5)	<0.001 *
**Hgb (g/dL)**	10.87 (9.1, 12.5)	9.89 (8.5, 11.2)	<0.001 *
**Platelet (10**^**3**^**/uL)**	139.0(69.0, 192.2)	105.3 (52, 131.2)	<0.001 *
**Prothombin Time (seconds)**	12.8 (11.3, 15.3)	16.5 (13.4, 21.4)	<0.001 *
**ALT (U/L)**	31 (19, 55)	36 (22, 75)	<0.001 *
**AST (U/L)**	47 (28, 88)	72 (41, 140)	<0.001 *
**Total bilirubin (mg/dL)**	1.3 (0.7, 2.7)	2.9 (1.2, 10.02)	<0.001 *
**Na (mEq/L)**	139 (136, 141)	138 (134, 141)	<0.001 *
**K (mEq/L)**	3.8 (3.4, 4.2)	3.7 (3.2, 4.3)	<0.001 *
**BUN (mg/dL)**	13.5 (9.0, 21.0)	24 (13.08, 46.00)	<0.001 *
**Creatinine (mg/dL)**	0.79 (0.61, 1.08)	1.09 (0.7, 2.2)	<0.001 *
**INR**	1.20 (1.09, 1.41)	1.55 (1.29, 2.0)	<0.001 *
**Albumin (g/dL)**	3.06 (2.58, 3.73)	2.60 (2.26, 2.97)	<0.001 *

Continuous data are reported as median (25th, 75th quatile) for non-normal distribution data and compared using the Mann-Whitney U Test; categorical data are shown as number (percentage) and compared using the Chi-square test.

* Indicates a significant difference between event and non-event groups.

Abbreviations: OR: Odds Ratio; HBV: hepatitis B virus; HCV: hepatitis C virus; SBP: spontaneous bacterial peritonitis; EV bleeding: esophageal variceal bleeding; WBC:white blood cell count; Hgb: hemoglobin; PT: prothrombin time; ALT: alanine transaminase; AST: aspartate transaminase; Na: serum sodium; K: serum potasium; BUN: Blood urine nitrogen; INR: international normalized ratio.

**Fig 1:**
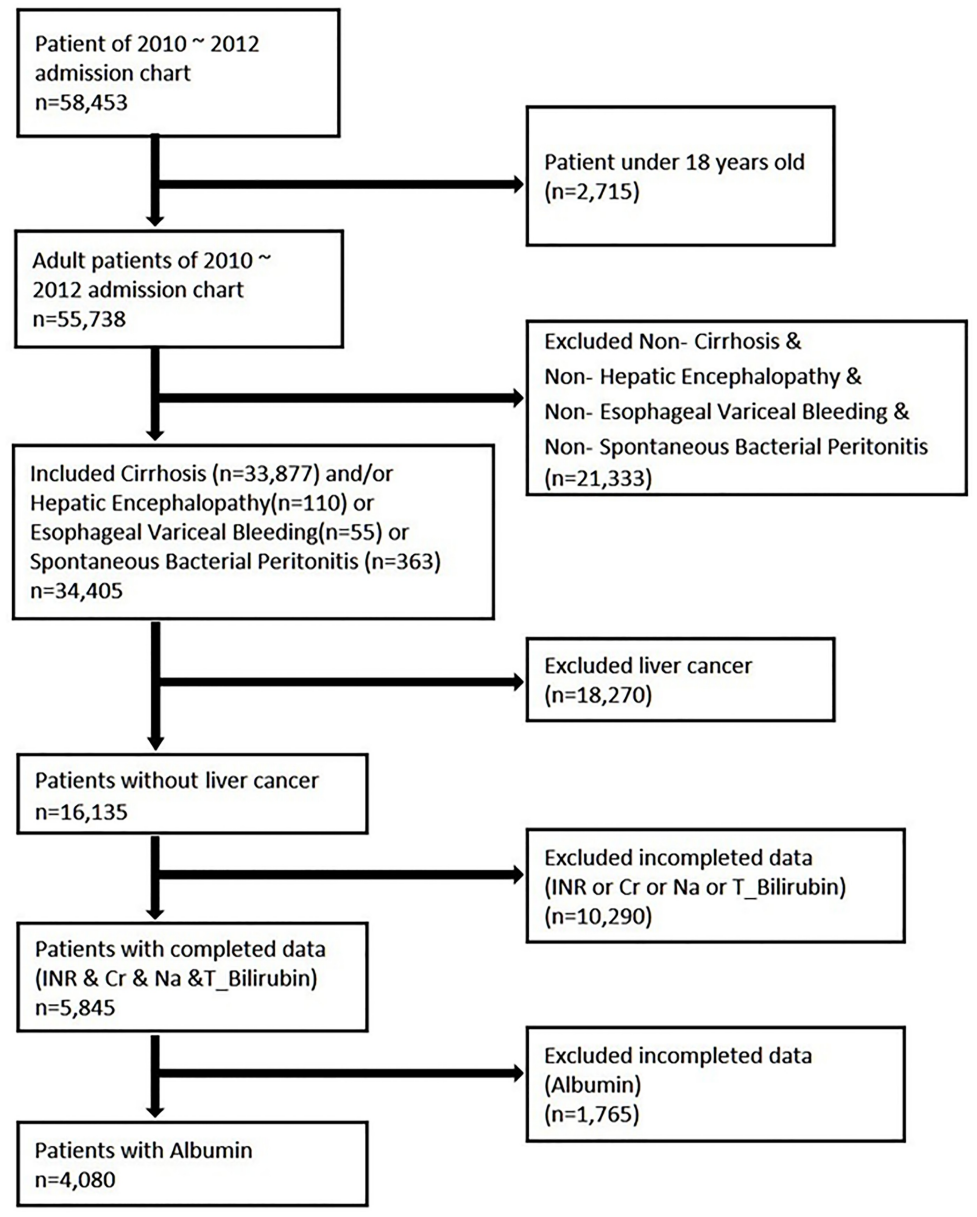
Flow chart of patients included in the study (n = 4080).

Fig 1 demonstrates the flow chart of the enrolment process.

### Baseline demographic and laboratory characteristics between derivation and validation data sets

Table 2 shows the baseline demographic and laboratory characteristics in derivation and validation data sets of non-cancer cirrhotic patients. No statistical differences were observed in age, sex, the percentage of chronic hepatitis B and chronic hepatitis C, and serum level of ALT, AST, total bilirubin, Na, BUN, Cr., and albumin between the derivation and validation data. The prevalence of complications also did not reach statistical differences between the two sets, except for the prevalence of sepsis (22.6% vs. 20.1% in derivation vs. validation sets, p < 0.001). However, the percentage of alcoholic liver cirrhosis were higher (35.9% vs. 31.6%, p = 0.001), and lower white blood cell counts (WBC), haemoglobin (Hgb), platelet counts, serum level of potassium, and INR were observed in the validation data than in the derivation set.

**Table 2:** Baseline demographic and laboratory characteristics of non-cancer cirrhotic patients in derivation and validation data sets.

Characteristics	Derivation set (n = 4080)	Validation set (n = 1875)	p Value
**Age (years)**	55 (44, 66)	55 (45, 66)	0.752
**Gender, n (%)**			0.415
Female	1261 (30.9)	600 (32)	
Male	2819 (69.1)	1275 (68)	
**Etiology, n (%)**			
HBV	896 (21.9)	392 (20.9)	0.377
HCV	716 (17.5)	336 (17.9)	0.755
Alcohol	1291 (31.6)	674 (35.9)	0.0011*
**Complicatios, n (%)**			
SBP	416 (10.2)	199 (10.6)	0.655
Hepatoencephalopathy	841 (20.6)	393 (20.9)	0.785
EV bleeding	1004 (24.6)	478 (25.5)	0.482
Sepsis	922 (22.6)	377 (20.1)	<0.001 *
**WBC (10**^**3**^**/uL)**	6.40 (4.40, 9.40)	6.10 (4.10, 8.90)	<0.002 *
**Hgb (g/dL)**	10.3 (8.9, 12.2)	10.1 (8.7, 11.9)	<0.006 *
**Platelet (10**^**3**^**/uL)**	103 (63, 175)	96 (57, 166)	<0.008 *
**PT (seconds)**	13.6 (11.68, 17)	13.8 (11.8, 16.5)	0.156
**ALT (U/L)**	32 (20, 59)	31 (19, 54)	0.068
**AST (U/L)**	53 (31, 101)	51 (31, 94)	0.313
**Total bilirubin (mg/dL)**	1.5 (0.8, 4.0)	1.5 (0.8, 3.6)	0.367
**Na (mEq/L)**	139 (135, 141)	138 (135, 141)	0.386
**K (mEq/L)**	3.8 (3.4, 4.2)	3.7 (3.2, 4.2)	0.003 *
**BUN (mg/dL)**	15.1 (10, 27.9)	15 (10, 29.6)	0.787
**Creatinine (mg/dL)**	0.84 (0.63, 1.29)	0.86 (0.64, 1.41)	0.098
**INR**	1.3 (1.1, 1.6)	1.29 (1.10, 1.50)	0.007 *
**Albumin (g/dL)**	2.90 (2.46, 3.50)	2.90 (2.50, 3.43)	0.879

Continuous data are reported as median (25th, 75th quatile) for non-normal distribution data and compared using the Mann-Whitney U Test; categorical data are shown as number (percentage) and compared using the Chi-square test.

* Indicates a significant difference between event and non-event groups.

Abbreviations: OR: Odds Ratio; HBV: hepatitis B virus; HCV: hepatitis C virus; SBP: spontaneous bacterial peritonitis; EV bleeding: esophageal variceal bleeding; WBC:white blood cell count; Hgb: hemoglobin; PT: prothrombin time; ALT: alanine transaminase; AST: aspartate transaminase; Na: serum sodium; K: serum potasium; BUN: Blood urine nitrogen; INR: international normalized ratio.

### Cox proportional hazard model for survival prediction of non-cancer cirrhotic patients

As shown in Table 3, the cox proportional hazard regression analysis was further performed after adjusting for the possible confounding variables, such as age, WBC, Hgb, platelet, PT, ALT, total bilirubin, Na, Cr., albumin, and INR. Age, albumin, total bilirubin, Cr., and INR were found to have a significant impact for survival prediction in non-cancer cirrhotic patients (hazards ratio [HR] 1.028, 95% confidence interval [CI]: 1.023–1.032; 0.602, 95% CI: 0.541–0.670; 1.304, 95% CI: 1.234–1.378; 1.404, 95% CI: 1.333–1.480; and 2.072, 95% CI: 1.895–2.266, respectively, all p < 0.001) in the cox proportional hazards model. Hence, the above variables were used in the construction of the novel model.

**Table 3:** Cox proportional hazard model for survival prediction of non-cancer end-stage liver disease.

	Hazard Ratio	95% CI	P value
Age	1.028	1.0231–1.0320	<0.001 *
Albumin	0.602	0.5414–0.6695	<0.001 *
Total bilirubin	1.304	1.2344–1.3777	<0.001 *
Creatinin	1.404	1.3327–1.4798	<0.001 *
INR	2.072	1.8952–2.2664	<0.001 *

Cox proportional harzards regression analysis for prediction of survival in non-cancer end stage liver disease. The model was adjusted for age, white blood cell counts, hemoglobin, platelet, prothrombin time, alanine transaminase, total bilirubin, sodium, creatinin, albumin, INR.

Abbreviations: CI: confidence interval; INR: international normalized ratio.

* Indicates a significant difference with a p value < 0.05

### Comparisons of discrimination ability among the novel score and the MELD and MELD-Na scores for non-cancer cirrhotic patients in the derivation set

**Fig 2:**
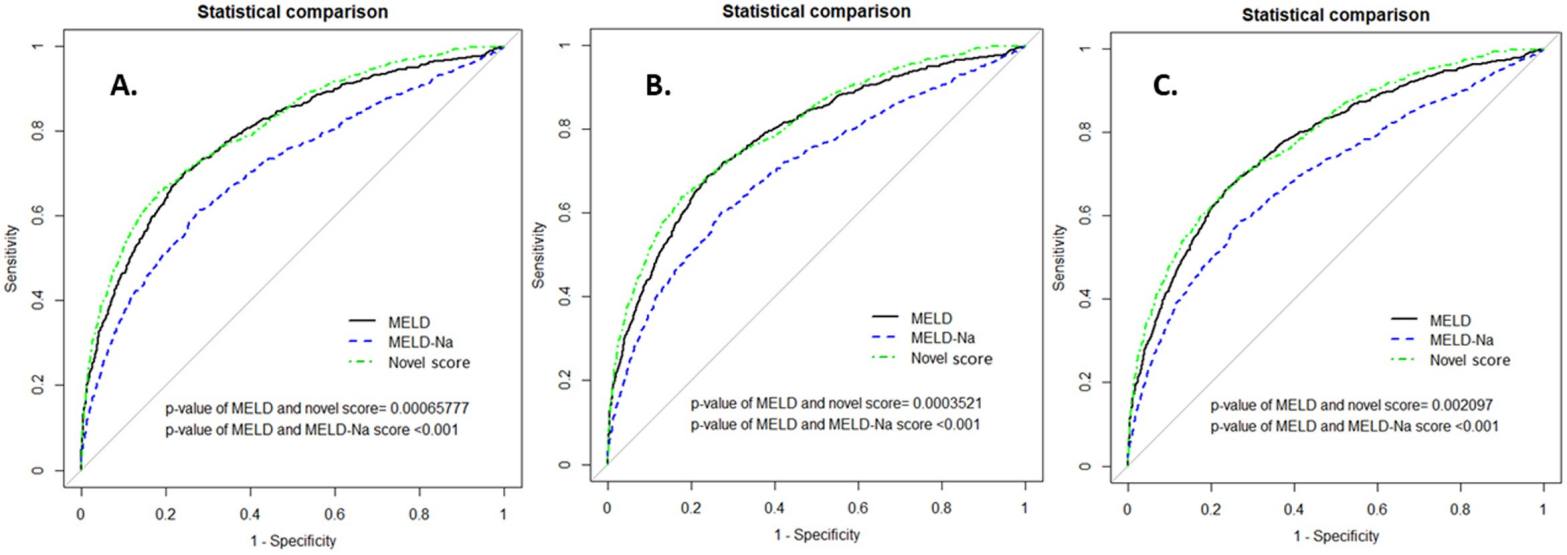
Comparisons of ROC curves of the 3-month, 6-month and at the end of follow-up mortality prediction among the novel score, MELD score, and MELD-Na score in non-cancer related cirrhotic patients. **A**. ROC curves of the 3-month mortality prediction among the three scores (n = 811). The AUC were 0.7873 (95% CI; 0.769–0.803), 0.7053 (95% CI; 0.684–0.727; *P<* 0.001), 0.8038 (95% CI; 0.786–0.821; *P*<0.001) in MELD, MELD-Na, and the novel model, respectively. **B**. ROC curves of the 6-month mortality prediction among the three scores (n = 887). The AUC were 0.7807 (95% CI; 0.763–0.799), 0.7023 (95% CI; 0.682–0.723; *P*<0.001), 0.7974 (95% CI; 0.780–0.814; *P*<0.001) in MELD, MELD-Na, and the novel model, respectively. **C**. ROC curves of at the end of follow-up mortality prediction among the three scores (n = 1188). The AUC were 0.7713 (95% CI; 0.754–0.789), 0.6937 (95% CI; 0.674–0.714; *P<* 0.001), 0.7852 (95% CI; 0.769–0.802; *P = 0*.*0021*) in MELD, MELD-Na, and the novel model, respectively.

To illustrate the performance of discrimination ability for the 28-day, 3-month, 6-month, and at the end of follow-up mortality in non-cancer cirrhotic patients, the AUROC was calculated. The performance of the novel model was further compared using the MELD and the MELD-Na scores in differentiating mortality predictions (see Table 4 and Fig 2). We found that the novel model had better discrimination than MELD and MELD-Na scores in the 3-month, 6-month and at the end of follow-up mortality prediction (AUROC of 3-month mortality of MELD vs. MELD-Na vs. novel score were 0.7873, 95% CI: 0.769–0.806 vs. 0.7053, 95% CI: 0.684–0.727, P<0.001 vs. 0.8038, 95% CI: 0.786–0.821, P<0.001; AUROC of 6-month mortality of MELD vs. MELD-Na vs. novel score were 0.7807, 95% CI: 0.763–0.799 vs. 0.7023, 95% CI: 0.682–0.723, P<0.001 vs. 0.7974, 95% CI: 0.780–0.814, P<0.001; AUROC of at the end of follow-up mortality of MELD vs. MELD-Na vs. novel score were 0.7713, 95% CI: 0.754–0.789 vs. 0.6937, 95% CI: 0.674–0.714, P<0.001 vs. 0.7852, 95% CI: 0.769–0.802, P = 0.0021). In addition, the AUROC of novel model in the 28-day mortality prediction were also better than MELD and MELD-Na. However, it had not reached the statistical significance. As current evidence showed, the performance of MELD score was the best in <1 month short-term mortality prediction in ESLD population.

**Table 4:** Comparisons of discrimination ability among the novel score, the MELD and MELD-Na scores for non-cancer cirrhotic patients in the derivation data set.

Scores	28 day mortality (n = 513)			3-month mortality (n = 811)			6-month mortality (n = 887)			At the end of follow up (n = 1188)		
	AUROC	95% confidence interval	p value vs. MELD	AUROC	95% confidence interval	p value vs. MELD	AUROC	95% confidence interval	p value vs. MELD	AUROC	95% confidence interval	p value vs. MELD
**MELD score**	0.8043	0.783–0.825		0.7873	0.769–0.806		0.7807	0.763–0.799		0.7713	0.754–0.789	
**MELD-Na score**	0.7096*	0.684–0.736	<0.001	0.7053*	0.684–0.727	<0.001	0.7023*	0.682–0.723	<0.001	0.6937*	0.674–0.714	<0.001
**Novel score**	0.8113	0.791–0.832	0.1891	0.8038*	0.786–0.821	<0.001	0.7974*	0.780–0.814	<0.001	0.7852*	0.769–0.802	0.0021

* Indicates a statistical significant difference (P < 0.05) compared to the MELD score.

Abbreviations: MELD: Model for End-Stage Liver Disease; MELD-Na: Model for End-Stage Liver Disease-Sodium; AUCROC, area under the curve of the receiving operating characteristic

### Calibration and external validation of novel score in non-cancer cirrhotic patients

**Fig 3:**
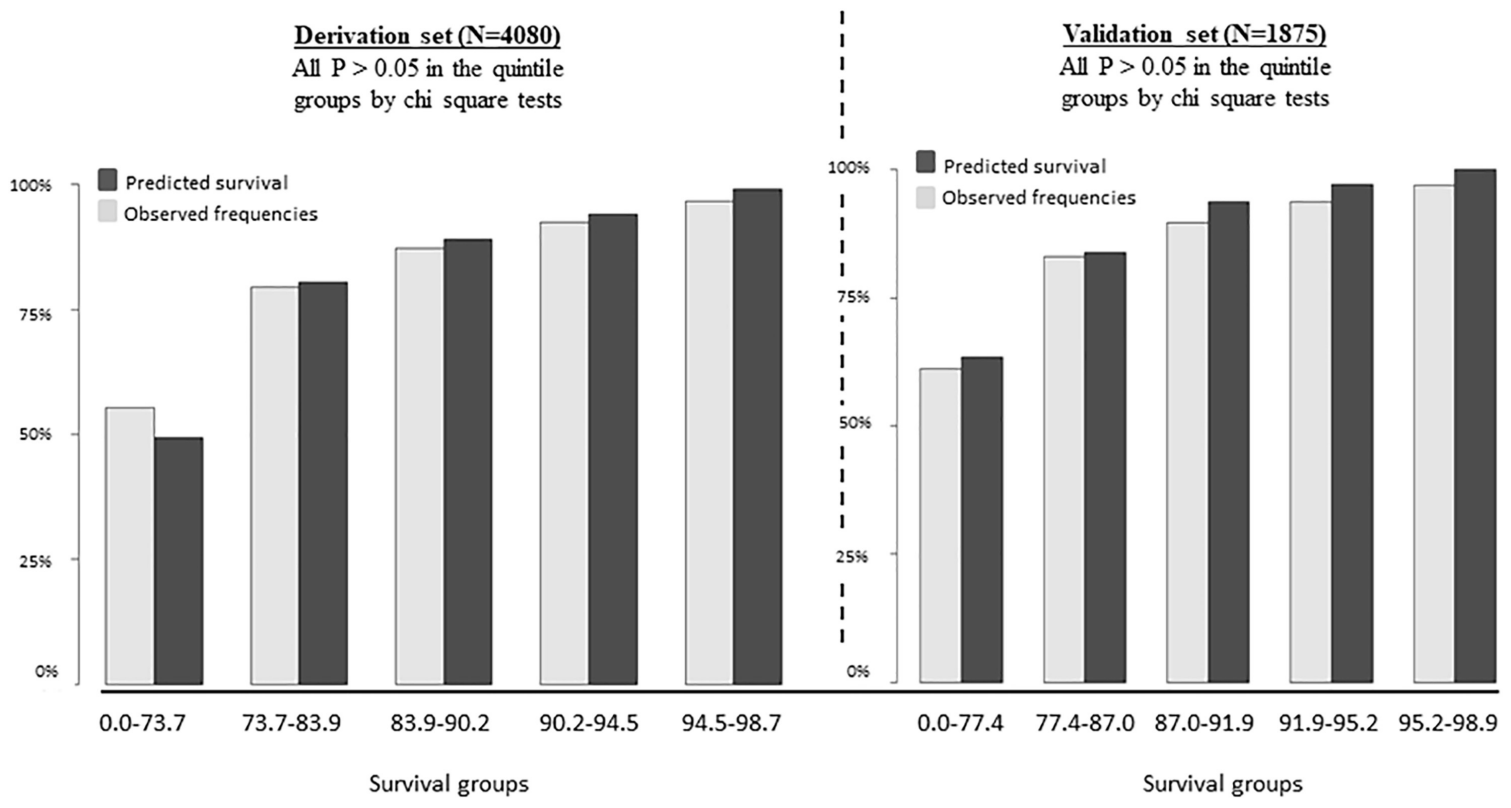
Approximate quintile of modified-MELD score distribution of the predicted vs. observed survival cases in the derivation and validation sets.

Fig 3 shows the calibration analysis of our novel score in both derivation and validation data sets to delineate whether it can predict equally well across the range of scores. All the quintile groups of the observed and the predicted data had not reached statistical differences by Chi-square tests in both derivation and validation data sets (p > 0.05).

We also performed the calibration of deciles of the novel score of the predicted vs. observed survival cases in the derivation and validation data sets. All the deciles of the novel scores of the observed and the predicted data had not reached statistical differences by Chi-square tests in both derivation and validation data sets (p > 0.05). (See *Supporting Information*).

## Discussion

This study was the first multicentre analysis to identify the 28-day, 3-month, 6-month and the overall prognostic score for non-cancer ESLD patients in Asian population. This was a pioneer study to develop a scoring system to early identify the survival prediction (especially for 6-month mortality prediction) in the relatively early stage or less severe cirrhotic patients in order to help the clinician in the clinical decision making for therapeutic management. The performance of novel score was superior to that of MELD and MELD-Na scores particularly in the 3-month, 6-month and at the end of follow-up survival prediction. (P<0.001 vs. P<0.001 vs. P = 0.002 respectively).

There were some valuable points of our novel score compared to the currently available scoring system (Child—Pugh, MELD, and MELD-Na). First, the parameters left in our novel score for estimating mortality were easy to obtain in standard hospitalization settings. Second, the novel score not only avoids clinician’s subjective judgement as the MELD and MELD-Na for survival analysis, but also provides better performance in 3-month, 6-month and overall prognosis prediction for early stage, less severe non-cancer related cirrhotic patients. Thereafter, the novel score helped to identify the prognosis earlier and aided the physician in clinical decision making for therapeutic management in less severe non-cancer related cirrhotic patients.

The calibration analysis of our novel scoring system in both derivation and validation data sets showed no statistical differences in both the observed and the predicted groups. In addition, the performance of the novel scoring system in predicting the 3-month, 6-month and at the end of follow-up survival was more statistically significant than the MELD and MELD-Na scores.

There were some limitations in this study. First, this was a retrospective cohort study design, and the subjects were mainly Taiwanese people, which may predispose some inherited or genetic variants in prognostic difference in races. Second, different aetiology of ESLD such as viral hepatitis or alcoholic causes, etc. or the aggravating factors which predisposed to acute on chronic liver failure was not further stratified to analyse in this study, and this may lead to some subgroup differences in mortality prediction during the clinical course[21, 22]. Furthermore, the therapeutic or intravenous drug administration was not documented in this study setting which might bear some potential interactions to the survival prediction of the score. At last, there were only <1% subjects undergoing liver transplantation which may be because the inclusion subjects were relatively less severe non-cancer cirrhosis. Hence, in regard to the relatively scarce percentage of liver transplantation, we did not include the competing risk of liver transplantation in the survival analysis.

To date, several prognostic tools have been developed for ESLD patients but all had limited utility or were suitable for certain situations such as liver transplantation or mortality prediction after transjugular intrahepatic portosystemic shunt, etc. Objective survival scoring systems developed for the majority of non-cancer terminal cirrhotic patients are scarce.

Unlike current published literatures, the MELD-Na score performed worse than MELD score in our study results. We raised some postulates. Because the enrolled patients in this study were relatively less severe (the median MELD vs. MELD-Na scores were 14.59 vs. 15.67), we found that age, albumin, total bilirubin, Creatinine, INR played more significantly influential part rather than serum Na on the mortality in the relatively early stage or less severe cirrhotic cases.

In our study, age, serum albumin, total bilirubin, Cr., and INR level during initial admission course were surrogates for prognosis in non-cancer ESLD patients. In the present study, we found that the lower the serum albumin level, the poorer the prognosis in non-cancer ESLD patients. In addition, the elderly and higher level of total bilirubin, Cr., and INR were poor prognostic factors. Compared with the previous prognostic tools in ESLD, our study demonstrated that albumin was the protective indicator of non-cancer ESLD survival. A study conducted by Xuan Zhu et al. demonstrated that a low serum albumin (<30 g/L) was the sole predictor for developing overt hepatic encephalopathy in covert hepatic encephalopathy patients[23]. Furthermore, overt hepatic encephalopathy increased the risk of hospitalization and death/transplant despite controlling for MELD[24]. The beneficial effect of serum albumin has been observed in our study. Albumin itself has a variety of important physiologic functions such as antioxidant effects, immunomodulation, and endothelial stabilization[25, 26] in addition to its traditional biologic and therapeutic role in liver disease attributed mainly to its oncotic effects. Therefore, human serum albumin, the important plasma protein produced by the liver with a number of accepted clinical indications in chronic liver disease, would be a clinically important indicator for prognosis prediction in non-cancer cirrhotic patients.

## Conclusions

In brief, the novel score is a clinically relevant, validated scoring system that can be used sequentially to stratify the prognosis in non-cancer cirrhotic populations, which may help clinical physicians in decision making of end-of-life care planning in the terminally ill non-cancer patients.

## Supporting information

S1 File The distribution, prognosis-predicted ability of the deciles of novel scores and calibration of deciles of the novel score for predicted v.s observed survival cases in the derivation and validation data sets. (PDF)

S2 File Dataset used in analyses. (XLS)
