# Supplementary material for: Survival prediction among patients with non-cancer-related end-stage liver disease
Source: PLoS One. 2018 Sep 21;13(9):e0202692. doi: 10.1371/journal.pone.0202692 (PMC6150508; doi:10.1371/journal.pone.0202692)

S1 File. The distribution, prognosis-predicted ability of the deciles of novel scores and calibration of deciles of the novel score for predicted v.s observed survival cases in the derivation and validation data sets

The median of the novel score was 70.27 (IQR: 60.45 – 80.58); the minimum was 43.40 and the maximum was 122.82.
The deciles of the novel score and their predicted ability about prognosis were as followed:

Deciles of novel score	1-month mortality prediction	3-month mortality prediction	6-month mortality prediction	Overall mortality prediction
< 55	0.67%	0.66%	0.64%	0.63%
55 ~ < 59	2.42%	2.56%	2.65%	2.71%
59 ~ < 63	4.83%	5.71%	5.76%	5,87%
63 ~ < 67	5.48%	11.88%	11.97%	11.95%
67 ~ < 71	11.20%	17.24%	17.34%	17.49%
71 ~ < 75	9.61%	12.12%	12.19%	12.22%
75 ~ < 79	9.56%	17.93%	17.94%	17.73%
79 ~ < 83	14.54%	23.28%	23.55%	23.60%
83 ~ < 90	28.00%	35.81%	35.82%	35.73%
≧ 90	49.65%	49.67%	49.66%	49.65%

The calibration of deciles of the novel score of the predicted v.s observed survival cases in the derivation data (N=4080)

X-squared = 0.032, p-value = 0.85>0.05

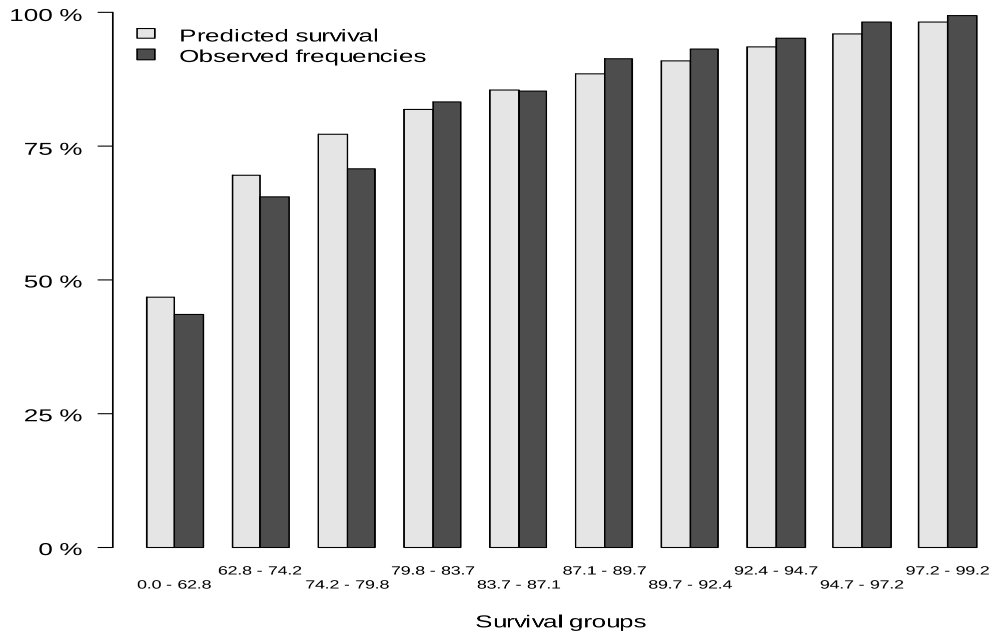

The calibration of deciles of the novel score of the predicted v.s observed survival cases in the validation data (N=1875)

X-squared = 0.496, p-value = 0.48 >0.05

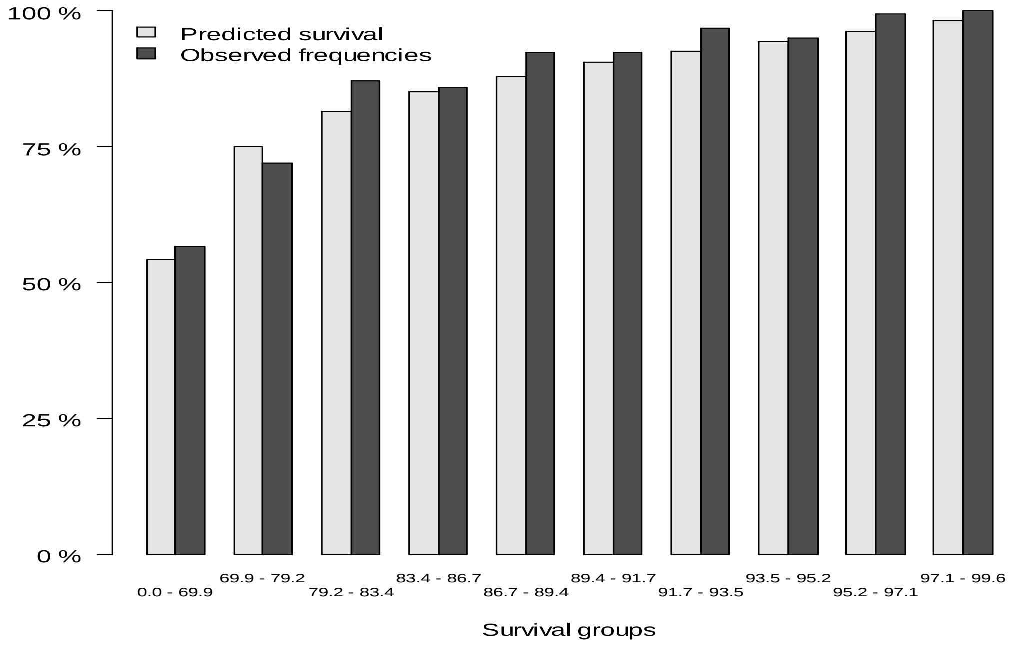

Supplement: S1 File — (PDF) [file pone.0202692.s001.pdf]
